# Association between uric acid and referable diabetic retinopathy in patients with type 2 diabetes

**DOI:** 10.1038/s41598-024-63340-0

**Published:** 2024-06-05

**Authors:** David Rivera-De-la-Parra, Sergio Hernández-Jiménez, Paloma Almeda-Valdés, Carlos A. Aguilar-Salinas, Enrique O. Graue-Hernández, Liliana Pérez-Peralta, Aida Jiménez-Corona, Group of Study CAIPaDi, David Rivera-De-la-Parra, Sergio Hernández-Jiménez, Denise Arcila-Martínez, Humberto Del Valle-Ramírez, Arturo Flores-García, Ana Cristina García-Ulloa, Mariana Granados-Arcos, Arely Hernández-Jasso, Diana Hernández-Juárez, Héctor Infanzón-Talango, Victoria Landa-Anell, Claudia Lechuga-Fonseca, Marco Antonio Melgarejo-Hernández, Angélica Palacios-Vargas, Liliana Pérez-Peralta, Francis Rojas-Torres, Sandra Sainos-Muñoz, Héctor Velázquez-Jurado, Andrea Villegas-Narváez, Luz Elena Urbina-Arronte, Carlos A. Aguilar-Salinas, Francisco J. Gómez-Pérez, David Kershenobich-Stalnikowitz

**Affiliations:** 1 Ophthalmology Department, Instituto de Oftalmología Conde de Valenciana IAP, Chimalpopoca 14, Colonia Obrera, Alcaldía Cuauhtémoc, 06800 Ciudad de México, México; 2 Centro de Atención Integral del Paciente Con Diabetes (CAIPaDi), Instituto Nacional de Ciencias Médicas y Nutrición Salvador Zubirán, México City, México; 3 Endocrinology Department, Instituto Nacional de Ciencias Médicas y Nutrición Salvador Zubirán, México City, México; 4 Dirección de Nutrición, Instituto Nacional de Ciencias Médicas y Nutrición Salvador Zubirán, Instituto Nacional de Ciencias Médicas y Nutrición Salvador Zubirán, México City, México; 5 General Directorate of Epidemiology, Health Secretariat, México City, México

**Keywords:** Microvascular disease, Uric acid, Diabetic retinopathy, Retinal diseases, Diabetes

## Abstract

Plasmatic uric acid (UA) has been inconsistently associated with diabetic retinopathy (DR). Specific sight-threatening stages of DR have not been studied for their association with UA. Cross-sectional, comparative study. Between 2014 and 2018 we recruited 210 Mexican individuals > 18 years-old with type 2 diabetes (T2D). Clinical, ophthalmological and biochemical assessment was performed with standardized funduscopic examination. Certified readers classified DR stages. The association between DR and UA was assessed by multiple logistic regression analysis, calculating odds ratios (OR) and 95% CI, after adjustment for covariates. Two hundred and ten patients were included, 41 (19.5%) had referable DR. Subjects with referable (severe or worse) DR had longer diabetes duration, 22 (15–28) vs 15 (8–20) years (*P* < 0.01); higher levels of UA, 6.5 (5.8–8.1) vs 5.4 (4.5–6.6) mg/dL (*P* < 0.01); higher systolic blood pressure, 130 (120–140) vs 120 (110–130) mmHg (*P* < 0.01); higher diastolic blood pressure, 78.4 ± 9.7 vs 75.4 ± 9.2 mmHg (*P* = 0.03); and lower glomerular filtration rate , 54.1 (41.5–69.6) vs 87.3 (66.8–108.3) mL/min/1.73m^2^ (*P* < 0.01) compared with those without referable DR. With multiple logistic regression, after adjustment, per each unit of change (mg/dL) in UA the probability of having referable DR increased 45% (OR = 1.45, 95% CI 1.12–1.87, *P* < 0.01). When UA was evaluated as dichotomous variable, those with levels ≥ 7.8 mg/dL had almost two times (OR = 2.81, 95% CI 1.00–7.9., *P* = 0.049) the probability of having referable DR compared with those with levels < 7.8 mg/dL. UA may contribute to the microvascular damage in retinal vessels and therefore hyperuricemia could be a therapeutic target to prevent DR progression.

## Introduction

Diabetic retinopathy (DR) is an important cause of visual loss and ocular morbidity, and a significant burden for the economy and public health^1–5^. Factors for DR have been studied in the past^6^. Some factors like glycated hemoglobin (HbA1c) and hypertension are completely established as important modifiable risk factors^7,8^.

Hyperuricemia is common in subjects with diabetes, and its role in the pathophysiology of microvascular complications of diabetes has been of interest^9^. A meta-analysis reported a significant association of hyperuricemia and the risk of peripheral neuropathy in patients with type 2 diabetes^10^. The association of uric acid (UA) with DR has controversial results^11–15^. A correlation has been described between the level of UA and worsening of DR^16,17^. Other studies did not find such association^14,15^. Kuwuata *et al.* described that the risk of developing DR was greater with higher levels of UA, where those in the fourth quartile had 2.17 times the probability of having DR compared with those in the first quartile^18^. Liang et al. reported an association between UA and DR with higher UA concentrations, having 1.26 times the likelihood of DR^19^.

Studies analyzing risk factors of DR usually determine the main outcome like the presence or absence of this complication, but severity has been not considered. For screening purposes and for the optimization of ophthalmological care, the severity of DR has been classified as referable or not referable, applying the Scottish diabetic retinopathy grading scale^20^. The use of referable retinopathy as the outcome may be of greater interest since subjects with this stage are more susceptible to visual loss^21^.

The aim of this study is to evaluate the association between UA concentrations and referable or sight threating retinopathy in patients with type 2 diabetes. Previous studies have not examined these outcomes thoroughly because they typically only take into account the presence or absence of DR without considering its stage. It is important to consider the stages in DR since individuals with more advanced disease have an increased risk of experiencing visual loss.

## Material and methods

A population-based cross-sectional study was carried out; charts of consecutive patients seen in the ophthalmology diabetes unit between 2014 and 2018 in a tertiary hospital (Instituto Nacional de Ciencias Médicas y Nutrición Salvador Zubirán) in México City were included. All patients were referred from the in-hospital diabetes clinic. We reviewed data of patients with type 2 diabetes, age 18 to 70 years-old, that were referred to our clinic for ophthalmic examination. Patients without an eye examination in the last year with any kind of therapy for hyperuricemia or nephropathy were also included. Exclusion criteria: Charts from patients with an incomplete ophthalmic examination, and patients with an eye pathology as cataract, cornea and optical media opacities that preclude an adequate ophthalmic diagnosis. Charts of patients with incomplete metabolic parameters were also excluded. Demographic data obtained from the chart for this study included age, gender, time of diagnosis of diabetes, and body mass index. Metabolic parameters obtained from the chart were taken with a maximum of three months from the ophthalmic examination and included UA levels, systolic blood pressure, diastolic blood pressure, fasting glucose, glomerular filtration rate (GFR) using the Modification of Diet in Renal Disease equation (MDRD), triglycerides, total cholesterol, high density lipoproteins, low density lipoproteins, and HbA1c.

In accordance with the Helsinki Declaration, all subjects were informed about the study objectives and provided written informed consent to participate. The Institutional Review Boards of Research, Ethics and Biosecurity from the Instituto Nacional de Ciencias Médicas y Nutrición Salvador Zubirán in México City approved the study protocol (ref 2454).

### Clinical examinations

Peripheral blood samples were taken after 8–12 h of fasting and measurements were carried out by using colorimetry with a Beckman Coulter equipment. UA concentration was determined in a period of less than 3 months from the date of ophthalmic examination. UA was analyzed both as a continuous variable and a dichotomous variable using  ≥ 7.8 mg/dL as threshold. The 7.8 mg/dL threshold was determined based on a series of logistic regression analyses where different values ranging from lower to higher were tested. The value of 7.8 mg/dL was found to have a significant association.

In the CAIPaDi clinic all ophthalmic examinations in patients with diabetes consist of the same standardized protocol, comprising visual acuity and pinhole visual acuity (PHVA) measurement taken with a standardized method using a LogMar chart (M&S technologies Inc. Smart system 20/20 Ilinois) at a 3-m distance; automatic refraction (Topcon KR-8900), and digital 5 field non-stereoscopic retinal photography with a non-mydiriatic camera (DRS, Padova, Italy) after pharmacological pupillary dilatation (fields centered in the macula, optic nerve, superior, inferior, and temporal to the macula). Cataract was documented using the slit lamp and following the Lens Opacities Classification System (LOCS II) scale.

### Grading of retinal photographs

Three standardized readers graded the retinal photographs. Previously, a subset of photographs of 60 eyes (by duplicate) was assessed for intra and interobserver variation, obtaining a kappa of 0.88. The photographs were evaluated in a masked manner to minimize any possible classification bias. The minimum criterion for the diagnosis of DR was the presence of at least one definite microaneurysm in any field photographed. DR was classified according to the International Diabetic Retinopathy Disease Severity Scale^22^.

### Primary outcome

The primary outcome was the presence of referable DR. It was defined by the presence of severe NPDR or a more advanced stage of DR in the worse eye according to the International Diabetic Retinopathy Disease Severity Scale^22^.

### Statistical analysis

Comparisons between those with and without referable DR was performed by using the student test or Kruskal–Wallis test, when appropriate. Proportions were compared with Pearson´s Chi-square test. Serum UA was analyzed both as a continuous and a dichotomous variable (< 7.8 and ≥ 7.8 mg/dL). The association between UA and referable DR was examined by logistic regression analysis, calculating odds ratios (OR) and their 95% CI, after adjustment for age (years), diabetes duration (years), systolic and diastolic blood pressure (mmHg), body mass index (kg/m^2^), fasting glucose (mg/dL), GFR (mL/min/1.73 m^2^), HDLcholesterol (mg/dL), non-HDL cholesterol (mg/dL), HbA1c (%), and hypertension (defined as taking antihypertensive therapy with previous medical diagnosis). The models were tested by Hosmer–Lemeshow goodness of fit; outliers and influence statistics were also evaluated. Due to the close relation of retinopathy and nephropathy, the regression models were repeated with normal (≥ 90 mL/min/1.73 m^2^) vs abnormal (< 90 mL/min/1.73 m^2^) GFR to prevent influence in the association results. All analyses were performed using STATA/MP 15.0 (Stata Corporation, College Station, TX, USA).

## Results

Of 1638 patients seen in the clinic, 210 patients met the inclusion criteria. Of these, 41 (19.5%) had referable DR. The demographic and anthropometric variables by DR groups are shown in Table 1. Subjects with referable DR had longer diabetes duration, higher levels of UA, higher systolic and diastolic blood pressure, and lower GFR compared with those without referable DR.

**Table 1:** Comparison of clinical characteristics between patients with and without referable diabetic retinopathy.

	Non-referable DR n = 169	Referable DR n = 41	*P* value
Age, years (median/IQR)	56(45–64)	57(45–63)	0.86
Gender female (subjects/%)	88 (52)	24 (58)	0.5
Diabetes duration, years (median/IQR)	15 (8–20)	22 (15–28)	** < 0.01**
Serum uric acid, mg/dL (median/IQR)	5.4 (4.5–6.6)	6.5 (5.8–8.1)	** < 0.01**
Systolic blood pressure, mmHg (median/IQR)	120 (110–130)	130 (120–140)	** < 0.01**
Dyastolic blood pressure, mmHg (mean ± SD)	75.4 ± 9.2	78.4 ± 9.7	0.03
Body mass index, kg/m^2^ (median/IQR)	27.6 (25–30.4)	29.0 (24.8–32.4)	0.15
Fasting glucose, mg/dL (median/IQR)	127.5 (197–176)	118 (88–147)	0.3
Glomerular filtration rate by MDRD equation, mL/min/1.73 m^2^ (median/IQR)	87.3 (66.8–108.3)	54.1 (41.5–69.6)	** < 0.01**
Glomerular filtration rate by MDRD equation ≥ 90, mL/min/1.73 m^2^ , (%)	80 (47)	3 (7)	** < 0.01**
Triglycerides, mg/dL (median/IQR)	133 (98–190)	135 (94–224)	0.8
Total cholesterol, mg/dL (median/IQR)	170 (145–200)	182 (156–209)	0.1
HDL-cholesterol, mg/dL (median/IQR)	44 (38–55)	49 (41–57)	0.08
Non-HDL cholesterol, mg/dL (median/IQR)	121 (98–154)	131 (107–154)	0.3
HbA1c, % and mmol/mol (median/IQR)	8.0 (7.2–9.5) 60 (55–80)	8.4 (7.1–10) 68 (54–86)	0.7

In an initial logistic regression (Table 2), after adjustment for age, diabetes duration, systolic and diastolic blood pressure, body mass index, fasting glucose, GFR, HDL-cholesterol, non-HDL cholesterol, hbA1c, and hypertension, per each unit of change (mg/dL) in UA, the probability of having referable DR increased 45% (OR = 1.45, 95% CI 1.12–1.87, *P* < 0.01). The logistic regression accounted for a positive association between the continuous value of UA and referable DR. After that initial analysis, different thresholds were tested; all values below 7.8 mg/dL resulted in a non-significant association, while 7.8 mg/dL and above showed significant values. When UA was evaluated as dichotomous variable, those with levels ≥ 7.8 mg/dL had more than two times (OR = 2.81, 95% CI 1.00–7.9, *P* = 0.049) the probability of having referable DR compared with those with levels < 7.8 mg/dL. Other significant associations were noted for diabetes duration, diastolic blood pressure, and GFR. In stratified models by GFR defined as normal (≥ 90 mL/min/1.73 m^2^) vs abnormal (< 90 mL/min/1.73 m^2^), results were statistically unchanged. The number of subjects with 7.8 mg/dL or above in the non-referable group was 28, and in the referable group it was 15.

**Table 2:** Logistic regression analysis for the association of UA and referable diabetic retinopathy.

	Unadjusted OR (95% CI)	*P* value	Model with UA in mg/dL OR (95% CI)	*P* value	Model with UA ≥ 7.8 mg/dL OR (95% CI)	*P* value
Serum uric acid (mg/dL)	1.56 (1.25–1.94)	< 0.01	1.45(1.12–1.87)	< 0.01	–	–
Serum uric acid ≥ 7.8 mg/dL	2.54(1.22–5.28)	0.01	–	–	2.81(1.00–7.90)	0.049
Age (years)	1.00(0.98–1.02)	0.62	–	–	–	–
Gender (male)	0.76 (0.38–1.53)	0.45	–	–	–	–
Diabetes duration (years)	1.06(1.03–1.10)	< 0.001	1.07(1.02–1.11)	< 0.01	1.06 (1.02–1.10)	< 0.01
Systolic blood pressure (mmHg)	1.02(1.00–1.03)	0.02	–	–	–	–
Diastolic blood pressure (mmHg)	1.03(0.99–1.07)	0.06	1.05 (1.00–1.10)	0.02	1.04 (1.00–1.09)	0.039
BMI (kg/m^2^)	1.04(0.97–1.12)	0.19	–	–	–	–
Fasting glucose (mg/dL)	0.99(0.99–1.00)	0.97	–	–	–	–
Glomerular filtration rate by MDRD equation, (mL/min/1.73 m^2^)	0.96(0.95–0.98)	< 0.001	0.98(0.96–0.99)	< 0.01	0.97 (0.96–0.99)	< 0.01
Triglycerides (mg/dL)	1.00(0.99–1.00)	0.52	–	–	–	–
Total cholesterol (mg/dL)	1.00(0.99–1.01)	0.12	–	–	–	–
HDL-cholesterol (mg/dL)	1.01(0.94–1.04)	0.23	–	–	–	–
Non-HDL cholesterol (mg/dL)	1.00(0.99–1.00)	0.18	–	–	–	–
HbA1c (%)	1.05(0.88–1.25)	0.53	1.17 (0.95–1.45)	0.12	1.14 (0.93–1.40)	0.18
Hypertension	3.78(1.58–9.01)	0.003	1.92(0.71–5.17)	0.19	2.14(0.80–5.67)	0.12

In the multiple logistic regression analysis, the variables age, systolic blood pressure, body mass index, fasting glucose, triglycerides, HDL-cholesterol, and non-HDL cholesterol showed no significant contribution to the association of UA and referable DR, and were excluded from the final regression model.

## Discussion

In this cross-sectional study, an increase in UA concentrations was independently associated with the presence of referable DR, defined as a DR stage of severe non-proliferative or worse. For a clinical scenario, our study showed that values of UA  ≥ 7.8 mg/dL were independently associated with an increased risk of referable DR. Other factors that were independently associated were duration of diabetes, diastolic blood pressure, and GFR. We have a positive hypothesis that links UA and DR. Even though it is not the risk factor with the highest odds ratio, it is still a modifiable risk factor that needs to be controlled. Proper control of UA may have a positive impact on microvascular outcomes.

Elevated UA is an important risk factor for progression of several lifestyle-related diseases, such as metabolic syndrome and type 2 diabetes that often have a common pathological basis^23^. Hyperuricemia is frequently present in patients with metabolic syndrome. UA levels are determined by a balance between its production and excretion. The enzyme responsible for its production (xanthine dehydrogenase / xanthine oxidase) is expressed in the liver, the parenchyma cells of the small intestine, adipose tissue, macrophages, and vascular endothelial cells^24^. This enzyme is also a major source of oxidative stress and generation of reactive oxygen species.

It is proposed that microvascular damage of UA may occur through inhibition of endothelial nitric oxide synthetase with a subsequent activation of the renin-angiotensin pathway^25^. UA may also cause damage to endothelial cells and induce changes on vascular smooth muscle cells^26^. One possible explanation for the coexistence of both elevated UA and DR is that both reflect advanced stages of diseases that may often be present together in patients with a poorly controlled metabolism. However, metabolism of UA may be linked to vascular endothelial cell damage and thus be directly related to the genesis and progression of DR.

Serum UA has been previously associated inconsistently with DR. Xia *et al.* found in a sample of 74 Chinese patients a higher concentration of UA among 39 subjects with DR and 34 without DR. In this study DR was classified as present or absent regardless of its degree or severity^15^. Lee *et al.* found in a sample of 749 Taiwanese subjects followed for 3 years an increase in the severity of DR in 13.8% of the sample. Progression of DR was associated with variables like duration of diabetes, HbA1c, albuminuria, and UA^14^. Values of UA associated with progression analyzed with Cox regression were those of the third (5.9–6.9 mg/dL) and fourth (≥ 7.0 mg/dL) quartiles, with a hazard ratio (HR) of 2.57 (95% CI 1.30–5.08) and 3.66 (95% CI 1.92–7.00), respectively, when compared with the first quartile (< 4.9 mg/dL).

Kuwata *et.al.* studied in a sample of 1839 Japanese patients the association of baseline UA with the incidence rate of DR at 2 years of follow-up. A significant HR was found in men in UA concentrations of the second (1.97 [95% CI 1.14–3.41], *P* = 0.015), third (1.92 [95% CI 1.18–3.13], *P* = 0.008), and fourth quartiles (2.17 [95% CI 1.40–3.37], *P* = 0.001) when compared with the first quartile. The association was not significant for women^18^.

A recent meta-analysis showed that UA levels were higher in subjects with DR, but this association was significant only in the late proliferative phase^27^. Our findings are consistent with these results because the outcome of referable DR that we used in our study can be considered an advanced stage. However, we included cases with severe non-proliferative DR and those with proliferative DR in our definition of referable DR. The authors of the meta-analysis considered this severe non-proliferative stage to be an early stage.

In 2021, a study was conducted on a Chinese population of 3481 individuals by Hu et al. The study found results similar to ours, which highlights a significant association between UA and Vision-threatening Diabetic Retinopathy (VTDR). The definition for DR used in their study was the same as ours. However, they utilized quartiles comparison for statistical analysis and found that individuals in the third and fourth higher quartiles had a significantly increased risk for VTDR^28^.

Previous studies have used different approaches in methods and statistical analysis for assessing the association between UA and DR. An important difference of our paper is that other studies used dichotomic definitions, i.e., presence or absence of DR, while in this paper we used referable DR vs non-referable DR as the main outcome. This stage of DR has more clinical impact since it is related to potential visual loss and thus should be considered as more useful in the clinical practice^29^.

In our study, values of 7.8 mg/dL and above showed significant association. These results suggest that higher levels of UA may be more suitable to be related to referable DR. Different to other studies, we did not use quartiles as a mean for comparison. An advantage of using the threshold of 7.8 mg/dL is that it allows for the clinician a more practical approach to apply in the clinical setting with the patient. The normal values for UA have not been officially established, but findings in our study suggest a value close to 7.8 to be associated with the specific outcome of referable DR.

Avci et al*.* described a significant association of elevated UA with DR adjusted for duration of diabetes (OR = 4.67, 95% CI 2.33–9.36). In our study the mean UA level was 5.4 mg/dL (IQR 4.5–6.6) in the group without DR and 6.5 mg/dL (IQR 5.8–8.1) in the DR group (*P* < 0.001). In the Avci et al*.* study, the main outcome was the presence of DR regardless of the severity, and no adjustment was made with other covariates^30^.

Limitations of our study are inherent to a cross-sectional design. Also, being a hospital-based population, it limits the external validity of the findings. No adjustment for the use of UA lowering agents or medical therapy for hyperglycemia or hypertension was included in the analysis. This could lead to an underestimation of the association in the case that therapy could have lowered the high levels of glycemia, hypertension and UA. This is not a longitudinal study, so associations suggest but do not confirm causality. Despite the limitations, some strengths are worth mentioning, namely that other variables for DR were available to include in the logistic regression model, accounting for a more precise association.

In conclusion, our study suggest that UA may contribute to the microvascular damage in retinal vessels and therefore hyperuricemia could be a therapeutic target to prevent DR progression, although further longitudinal studies are needed to confirm or discard the importance of its treatment in the pathogenesis of DR.

### Supplementary Information

Supplementary Information 1.

## Data Availability

We uploaded the date in supplementary materials.
